# Supplementary figures and images for: Comparison of the kinetics and magnitude of antibody responses to different SARS-CoV-2 proteins in Sinopharm/BBIBP-CorV vaccinees following the BNT162b2 booster or natural infection
Source: PLoS One. 2022 Oct 13;17(10):e0274845. doi: 10.1371/journal.pone.0274845 (PMC9560485; doi:10.1371/journal.pone.0274845)

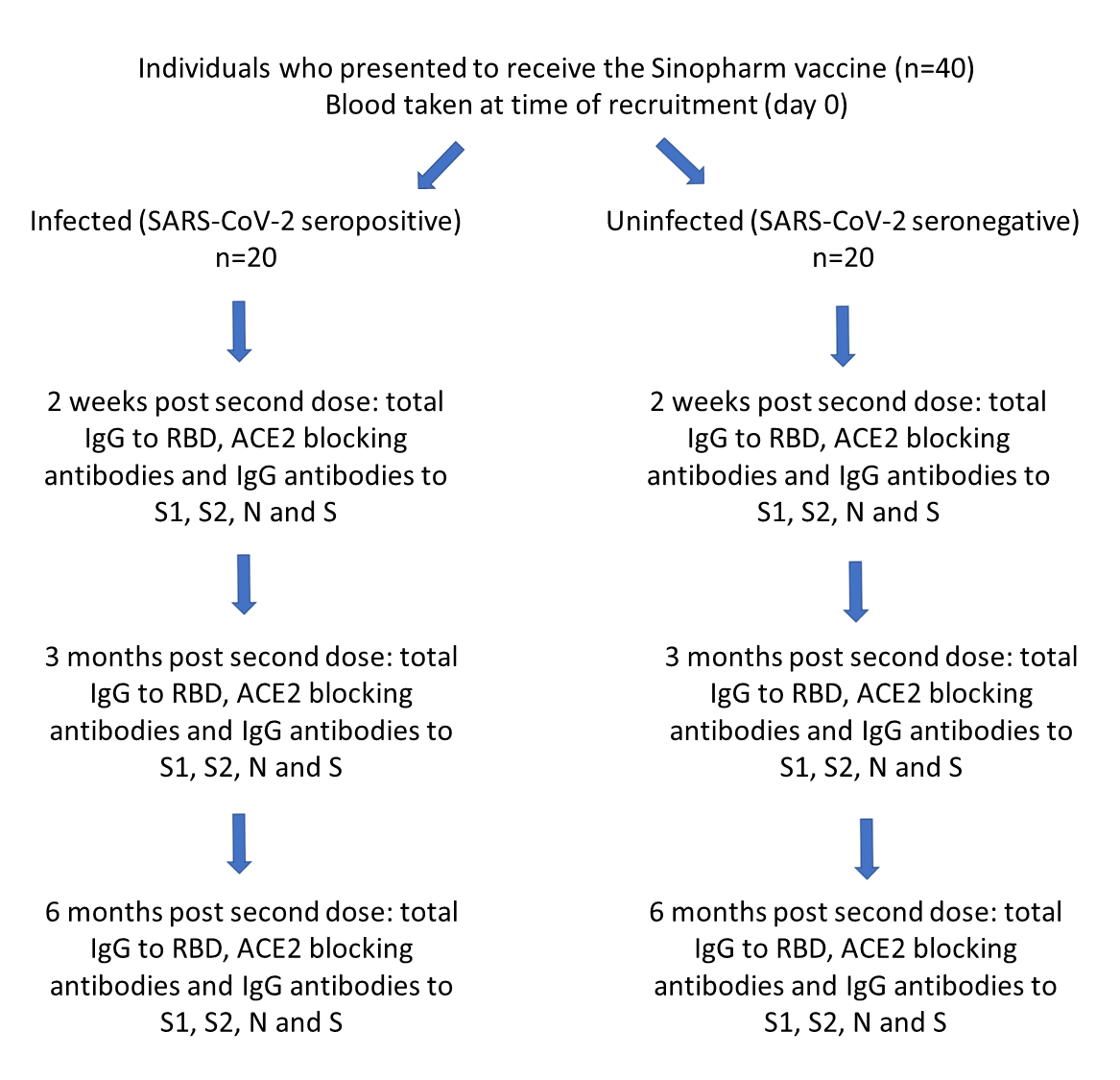

Supplement: S1 Fig — (TIF) [file pone.0274845.s001.tif]

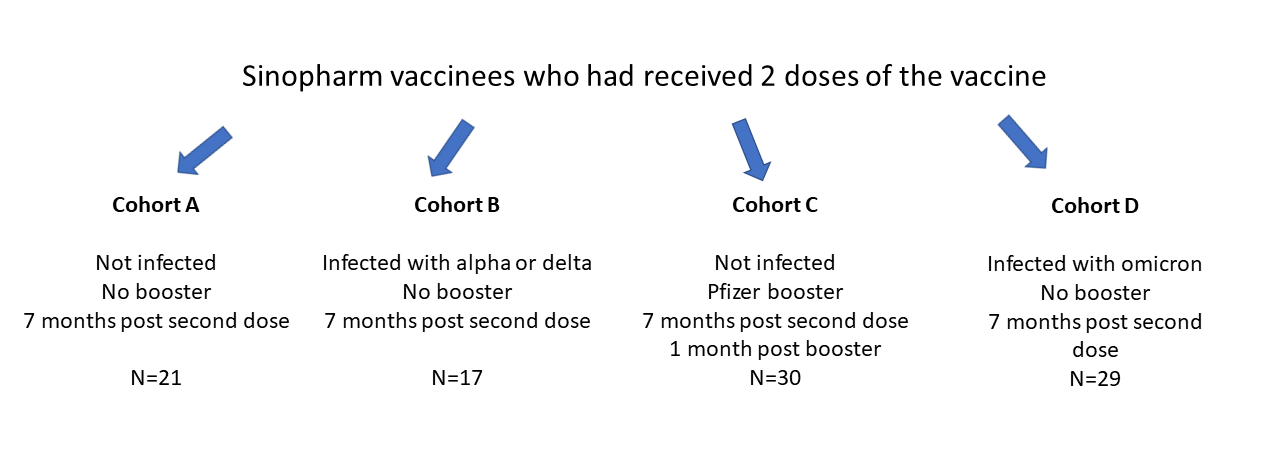

Supplement: S2 Fig — (TIF) [file pone.0274845.s002.tif]

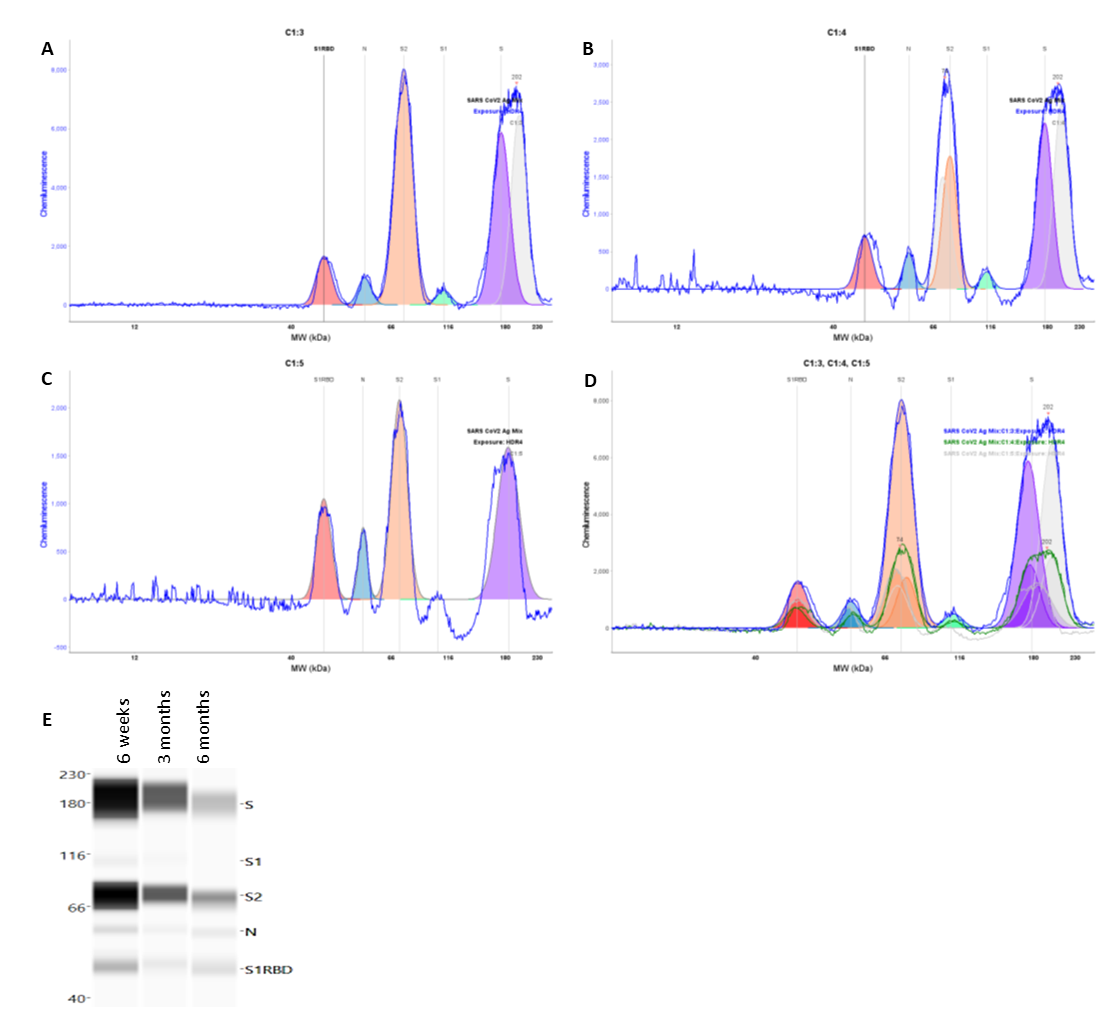

Supplement: S3 Fig — (A-D) Graph images obtained from Compass SW. (E) Lane view in Compass SW. (A) at 6 weeks (B) at 3 months (C) at 6 months (D) Overlayed graph of A, B and C (E) Lane view of 6 weeks, 3 months, and 6 months of the same individual. (TIF) [file pone.0274845.s003.tif]

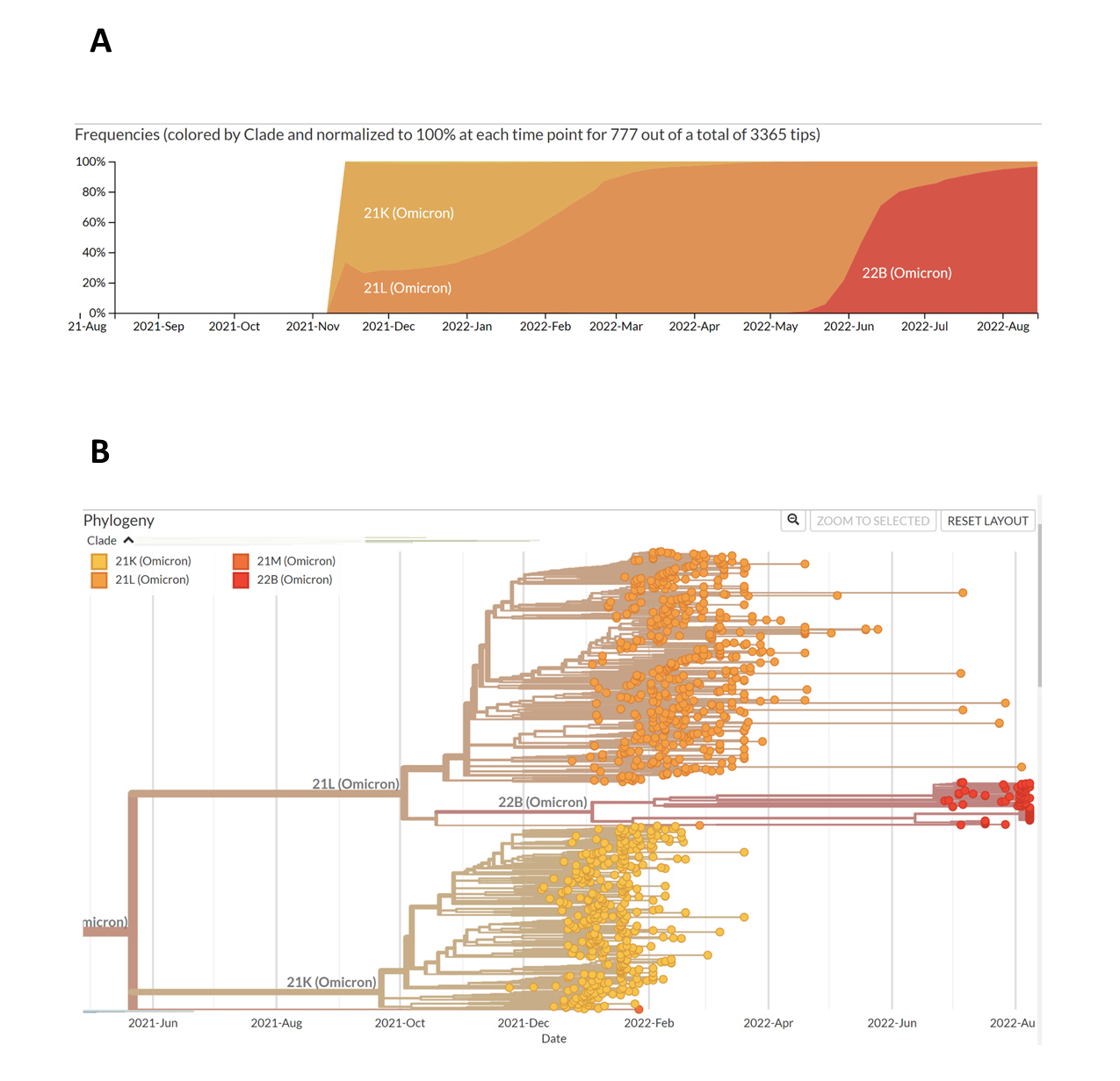

Supplement: S4 Fig — The frequency (A) and phylogenic tree (B) of the SARS-CoV-2 variants identified in Sri Lanka from December 15th onwards. All sequencing data of our laboratory was uploaded to GISAID and the figures were obtained from Nextstrain [11]. (TIF) [file pone.0274845.s004.tif]
